# Novel application of automated machine learning with MALDI-TOF-MS for rapid high-throughput screening of COVID-19: a proof of concept

**DOI:** 10.1038/s41598-021-87463-w

**Published:** 2021-04-15

**Authors:** Nam K. Tran, Taylor Howard, Ryan Walsh, John Pepper, Julia Loegering, Brett Phinney, Michelle R. Salemi, Hooman H. Rashidi

**Affiliations:** 1 Department of Pathology and Laboratory Medicine, University of California Davis, 4400 V St., Sacramento, CA 95817 USA; 2 Shimadzu North America/Shimadzu Scientific Instruments, Inc., Baltimore, USA; 3 Spectra Pass, LLC and Allegiant Airlines, Las Vegas, USA

**Keywords:** Clinical microbiology, Infectious-disease diagnostics, SARS-CoV-2, Machine learning

## Abstract

The 2019 novel coronavirus infectious disease (COVID-19) pandemic caused by severe acute respiratory syndrome coronavirus 2 (SARS-CoV-2) has created an unsustainable need for molecular diagnostic testing. Molecular approaches such as reverse transcription (RT) polymerase chain reaction (PCR) offers highly sensitive and specific means to detect SARS-CoV-2 RNA, however, despite it being the accepted “gold standard”, molecular platforms often require a tradeoff between speed versus throughput. Matrix assisted laser desorption ionization (MALDI)—time of flight (TOF)—mass spectrometry (MS) has been proposed as a potential solution for COVID-19 testing and finding a balance between analytical performance, speed, and throughput, without relying on impacted supply chains. Combined with machine learning (ML), this MALDI-TOF-MS approach could overcome logistical barriers encountered by current testing paradigms. We evaluated the analytical performance of an ML-enhanced MALDI-TOF-MS method for screening COVID-19. Residual nasal swab samples from adult volunteers were used for testing and compared against RT-PCR. Two optimized ML models were identified, exhibiting accuracy of 98.3%, positive percent agreement (PPA) of 100%, negative percent agreement (NPA) of 96%, and accuracy of 96.6%, PPA of 98.5%, and NPA of 94% respectively. Machine learning enhanced MALDI-TOF-MS for COVID-19 testing exhibited performance comparable to existing commercial SARS-CoV-2 tests.

## Introduction

The 2019 novel coronavirus infectious disease (COVID-19) pandemic caused by severe acute respiratory syndrome coronavirus 2 (SARS-CoV-2) has created a significant demand for testing^1,2^. In the United States, delays in establishing high throughput testing capacity early in the pandemic and subsequent supply shortages limited the nation’s ability to control the spread of COVID-19^3,4^.

SARS-CoV-2 diagnostic testing relies on molecular or antigen platforms (Table 1)^5^. Molecular methods such as reverse transcription (RT) polymerase chain reaction (PCR) offers highly sensitive and specific means to detect SARS-CoV-2 RNA. Unfortunately, despite molecular technologies serving as the accepted “gold standard” for SARS-CoV-2 diagnostics, these techniques are often dependent on constrained supplies chains (e.g., molecular grade reagents and consumables, processing plates/pipettes, extraction kits, etc.)^6^. Moreover, molecular platforms typically tradeoff between speed versus throughput^6,7^. Rapid (< 20 min) point-of-care molecular platforms, for example, are often being limited to testing one sample at a time, while high throughput laboratory-base instruments tests in batches every few hours—realistically producing results within 24–48 h. Antigen testing offers a unique alternative to molecular diagnostics by detecting SARS-CoV-2 proteins rather than RNA^8,9^. However, current data suggests antigen methods exhibit lower sensitivity and specificity especially when testing asymptomatic populations. *To this end, there is a critical need for a highly sensitive and specific high throughput method that is cost-effective, and rapid, for screening COVID-19.*

**Table 1:** Comparison of common emergency use authorized diagnostic methods for evaluating COVID-19 tests.

A. Molecular assays						
Manufacturer/Platform	Method	Throughput	RNA targets	LoD (NDU/mL)	PPA (%)	NPA (%)
Abbott Molecular/Alinity m	RT-PCR	300 test/8 h^a^	N1/N2	2700	> 99.0	> 99.0
Becton Dickenson/BDMax	RT-PCR	12 tests/3 h	N1/N2	1800	> 99.0	> 99.0
BioFire Defense/FilmArray	RT-PCR	1 test/45 min	ORF1ab/ORF8	5400	> 99.0	> 99.0
Bio-Rad/QX200	ddPCR	96 test/6 h	N1/N2	600	> 99.0	> 99.0
CDC/ABI 7500	RT-PCR	21 test/5 h	N1/N2	18,000	> 99.0	> 99.0
Cepheid/GeneXpert	RT-PCR	1–16 tests/1 h^b^	N2/E	5400	> 99.0	> 99.0
Hologic/Panther Fusion	RT-TMA	500 tests/8 h	ORF1ab	600	> 99.0	> 99.0
Thermo Fisher/Amplitude	RT-PCR	3000 tests/24 h	ORF1ab/N/S	180	> 99.0	> 99.0
Roche Molecular systems/cobas 6800	RT-PCR	94 tests/3 h	ORF1ab/E	1800	> 99.0	> 99.0
Roche Molecular systems/cobas Liat	RT-PCR	1 test/20 min	ORF1ab/N	5400	> 99.0	> 99.0

B. Antigen assays						
Manufacturer/platform	Method	Ag targets	LoD (TCID50/mL)	PPA (%)	NPA (%)	
Abbott Diagnostics/BinaxNow COVID-19 Ag	ICMA	N	140.6	84.6	98.5	
AccessBio/CareStart COVID-19 Ag	ICMA	N	6.4 × 10^3^	83.3	100.0	
Becton Dickenson/Veritor	IMCA	N	1.4 × 10^2^	84.0	100.0	
Ellume Limited/Elumme COVID-19 home	LFIA	N	1.0 × 10^3.8^	91.0	96.0	
Lumira Dx/LumeriaDx SARS-CoV-2 Ag test	LFIA	N	2.8 × 10^5^	97.6	96.6	
Quidel/Sofia-2	LFIA	N	3.4 × 10^5^	96.7	100.0	

*ABI* applied biosciences, *Ag* antigen, *CDC* Centers for Disease Control and Prevention, *ddPCR* digital droplet PCR, *E* envelope protein gene, *IMCA* immunochromographic membrane assay, *LFIA* lateral flow immunofluorescent assay, *LoD* limit of detection, *N* nucleoprotein gene, *NDU* nucleic acid test detectable units, *NPA* negative percent agreement, *ORF* open reading frame, *PCR* polymerase chain reaction, *PPA* positive percent agreement, *RNA* ribonucleic acid, *RT* reverse transcription, *S* spike protein gene, *TCID50* median tissue culture infective dose, *TMA* transcription mediated amplification.

^a^Random access capable.

^b^Instrument model dependent.

Matrix assisted laser desorption ionization (MALDI)—time of flight (TOF)—mass spectrometry (MS) has been proposed as a potential solution for COVID-19 testing^10^. Briefly, MALDI-TOF-MS has been employed in clinical microbiology over the last decade to accelerate identification of bacterial and fungal species from positive culture samples^11,12^. The technique produces mass spectra that represents ionizable protein components found in the sample that may correspond with a pathogen and/or disease state. For any given sample, there could be numerous mass spectra peaks perhaps hundreds or thousands—making analysis of complex samples or diseases challenging. Current advances in machine learning approaches complements and enhances performance of these MS-based technologies to analyze these complex samples. In this paper, we describe a proof-of-concept novel automated machine learning (ML) enhanced MALDI-TOF-MS approach for testing nasal swabs from patients with suspected COVID-19. An automated ML platform was used for data analysis.

## Methods

We conducted a bench analytic study to evaluate the performance of the MALDI-TOF-MS COVID-19 testing method using SARS-CoV-2 RNA PCR positive and negative samples. The goal of this study was to determine the accuracy along with the positive percent agreement (PPA) and negative percent agreement (NPA) of the MALDI-TOF-MS method to the PCR method that was used as a comparative approach.

### Study population/samples

The study was approved by the UC Davis Institutional Review Board. Informed consent was obtained was obtained for 226 nasal swab samples (anterior nares) preserved in saline transport media were obtained from the UC Davis Clinical Laboratory Biorepository. All methods were carried out in accordance with relevant guidelines and regulations. Patients included asymptomatic and symptomatic populations including those meeting COVID-19 testing criteria (i.e., patients who presented with or without COVID-19 and/or influenza-like illness symptoms at the time of collection) as well as asymptomatic apparently healthy volunteers as part of workplace screening. Saline viral transport media was used due to its widespread availability and compatibility with MALDI-TOF-MS techniques. Commercially available swabs (Copan, Murrieta, CA) were used for collection. All samples were stored at − 70 °C prior to testing.

### MALDI-TOF-MS method

**Figure 1:**
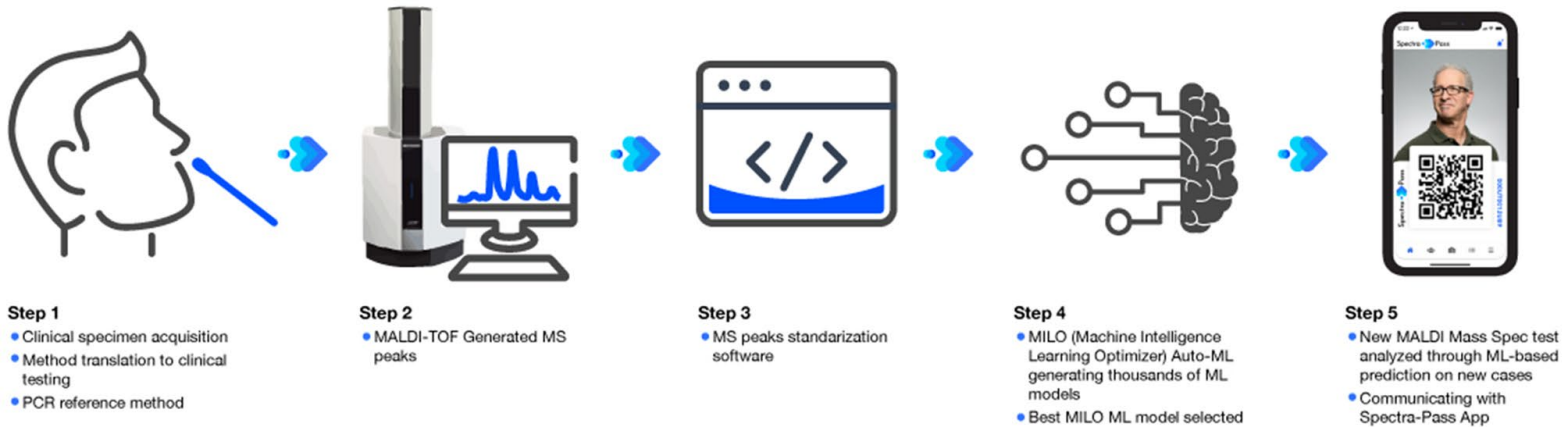
Conceptual drawing of study workflow. The study workflow consisted of patients providing a nasal swab specimen preserved in saline transport media. Media was tested by RT-PCR (Step 1) and swabs plated onto the MAALDI-TOF–MS platform (Step 2). Mass spectra were standardized (Step 3) and then analyzed using machine learning via the Auto-ML MILO platform (Step 4). COVID-19 status is then exported to a smart device app (Step 5).

The study testing workflow is illustrated by Fig. 1. Mass spectrometry testing was performed on a Shimadzu 8020 (Shimadzu Scientific Instruments, Columbia, MD) MALDI-TOF-MS analyzer. Sample processing was conducted under a Class II Biosafety Cabinet. Nasal swabs were removed from their respective saline transport media and first plated directly onto the MALDI-TOF-MS target plate. The tip of the swab was lightly tapped onto the target plate by feel to produce a ~ 1 μL drop in the well. After the swab was plated, this was followed by addition of 1 μL mixture of α-cyano-4-hydroxycinnamic acid (CHCA), ethanol, acetonitrile, and water solution with 3% trifluoroacetic acid (TFA). The use of CHCA was based on known performance and prior MALDI COVID-19 publications in this area^10,13,14^. To prepare the matrix solvent, mix 3.3 mL of high-performance liquid chromatography (HPLC) grade acetonitrile, 3.3 mL HPLC grade ethanol and 3.3 mL of deionized high purity water (i.e., Milli-Q or HPLC grade). To this, add 300 mL of trifluoroacetic acid (TFA). Carefully mix the solution. Then weight out 10 mg of CHCA into a 1.5 mL microcentrifuge tube and add 1 mL of the matrix solvent for a final concentration of 10 mg/mL.

Plated samples were then inactivated by ultraviolet (UV) irradiation for 10 min for inactivation of pathogens on the MALDI-TOF-MS plate. Thereafter, the target plate was transferred to the MALDI-TOF-MS analyzer for testing. MALDI-TOF-MS settings included a mass range of 2000–20,000 Daltons. Ten laser shots were fired for each profile at a frequency of 100 Hz using a dithering pattern (total of 1000 shots per well) and Gaussian smoothing method. Post-acquisition baseline subtraction and smoothing was performed using MALDI Solutions software (Shimadzu Scientific Instruments, Columbia, MD) (parameters: Baseline Filter Width = 250, Smoothing = Gaussian, and Smoothing Width = 50, Peak Width = 5). Peak picking was also performed by MALDIQuant software (Shimadzu Scientific Instruments, Columbia, MD). Threshold Apex algorithm was used for peak selection where the peak mass is assigned by selecting the highest point on the peak. Based on this protocol, the MALDI-TOF-MS would complete 48 runs (samples and quality control) every 20 min. Mass spectra were then standardized prior to analysis by ML with peak selection/alignment performed using MALDIQuant software.

### Comparative method

Residual saline transport media was tested by RT-PCR using Food and Drug Administration (FDA) emergency use authorized (EUA) assays (Table 1)^5^. These EUA assays included the cobas 6800 (Roche Molecular Systems, Pleasanton, CA), and digital droplet RT-PCR (Bio-Rad, Hercules, CA). Briefly, the cobas 6800 SARS-CoV-2 EUA assay targets open reading frame 1ab (ORF1ab) and envelope protein (E) gene regions, while the digital droplet RT-PCR method targeted two regions within the nucleocapsid (N) protein region. Both assays report sensitivity and specificity of > 99% based on their FDA EUA documentation. The use of two different assays was due to supply constraints during the pandemic.

### Machine learning

**Figure 2:**
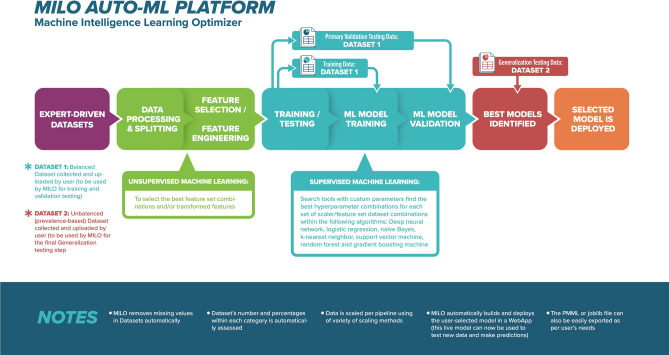
Machine intelligence learning optimizer Fig. 1. The MILO auto-machine learning (ML) infrastructure consists of beginning with two datasets: (**a**) balanced data (Dataset A) set used for training and initial validation, and (**b**) an unbalanced dataset (Dataset B) for generalization/secondary testing. MILO initially removes the missing values followed by providing several scaling options for the given dataset which is then assessed by the software. Unsupervised ML is then used for feature selection and feature engineering. The generated models are then trained on a subset (80%) of dataset A (depicted as Dataset 1 in the image above) and then initially tested with the remaining subset (20%) of Data Set A during its supervised ML stage. Following this training/initial validation stage, each of the ML models generated in this stage are then secondarily tested on Dataset B (depicted as Dataset 2 in the image above) for generalization testing. Selected models can then be deployed thereafter as joblib files. For this study, we imported the study data into MILO using COVID-19 status as the outcome measure for analysis. The following functions are then performed automatically by MILO.

The machine learning (ML) aspects of this study were carried out through the Machine Intelligence Learning Optimizer (MILO) automated ML platform (MILO ML, LLC, Sacramento, CA) which has been published in several recent papers^15–18^. Briefly, MILO includes an automated data processor, a data feature selector (ANOVA F select percentile feature selector and RF Feature Importances Selector) and feature set transformer (e.g., principal component analysis), followed by its custom supervised ML model builder using its custom hyperparameter search tools (i.e., its custom grid search along with its random search tools) to help find the optimal hyperparameter combinations within the variety of its embedded supervised algorithms/methods (i.e., deep neural network [DNN], logistic regression [LR], naïve Bayes [NB], k-nearest neighbor [*k-*NN], support vector machine [SVM], random forest [RF], and XGBoost gradient boosting machine GBM]). Ultimately, MILO employs a combination of unsupervised and supervised ML platforms from a large set of algorithms, scalers, scorers and feature selectors/transformers to create thousands of unique ML pipelines (Fig. 2) that generates over a hundred thousand models that are then statistically assessed to ultimately identify the best performing model for one’s given task.

For this study, we imported the trial data into MILO using COVID-19 status as the outcome measure for analysis. The aforementioned functions were then performed automatically by MILO. Information is assessed to ensure model training and the initial validation step is based on a balanced dataset. Initially in the build phase of MILO, the first balanced Dataset A is split into training and validation test sets in an 80–20 split with a 10 k-fold cross validation step, respectively. Since many algorithms benefit from scaling, in addition to using the unscaled data, the training dataset also underwent two possible scaling transformations (i.e., standard scaler and minmax scaler). To evaluate the effect of different features within the datasets on model performance, a combination of various statistically significant feature subsets (i.e., various MS peaks) or transformed feature sets were also selected to build new datasets with less features or transformed feature sets to feed into the various aforementioned supervised algorithms. The features selected in this step are derived from several well-established unsupervised ML/statistical techniques including ANOVA F-statistic value select percentile, RF Feature Importances or transformed using its principal component analysis approach^9^. A large number of supervised machine learning models are then built through this approach from these datasets with optimal parameters through MILO’s various supervised algorithms (i.e., DNN, SVM, NB, LR, *k*-NN, RF, and GBM), scalers, hyper-parameters, and feature sets. Notably, the final validation of each model within MILO is not based on the 20% test set mentioned earlier that generated from the initial training dataset (i.e., Dataset A) but rather each ML model’s true performance is based on its predictive capability on the independent secondary dataset (Dataset B). Ultimately, for final model validation, MILO’s thousands of generated models are then individually passed onto this next phase of the MILO engine generalization assessment phase (Fig. 2). This secondary testing approach markedly reduces the possibility of overfitted ML models since the model’s final performance measures are based on an independent secondary dataset only (Dataset B) as noted above. The final machine learning model performance data results are then tabulated by MILO’s interface and reported as clinical sensitivity, specificity, accuracy, negative predictive value (NPV), positive predictive value (PPV), F1 score, receiver operator characteristic (ROC) curves, and brier scores with reliability curves.

### Statistical analysis

Statistical analysis was performed using JMP Software (SAS Institute, Cary, NC). Area under the ROC curve analysis was also performed, as well as calculating PPA and NPA which served as surrogates for sensitivity and specificity. The use of PPA and NPA is recommended by the FDA due to not having a proven “gold standard” for SARS-CoV-2 detection at this time^5,19^. An independent Principal component analysis (PCA) within scikit learn was also performed on the greater than 600 MS peaks evaluated here and it’s PC1, 2 and 3 components (results not shown) highlighted many of the shared peaks noted within the MILO feature selector approach (i.e., RF Importances features [25%]) that found one of the best performing ML models for this study.

## Results

### Study population

**Figure 3:**
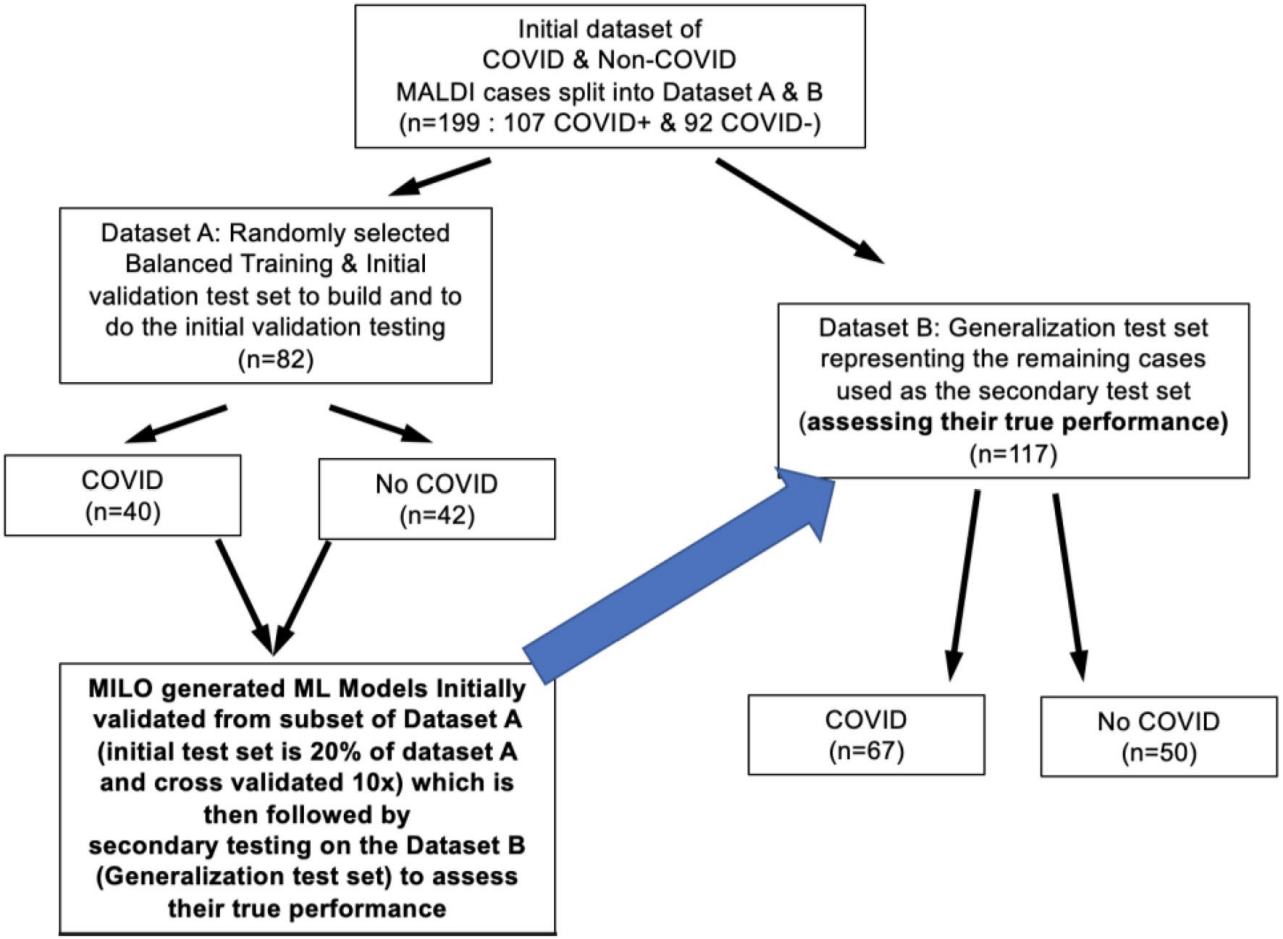
Study datasets. A total of 226 asymptomatic and symptomatic patients were enrolled. Twenty-seven samples were invalid due to polymer contamination, preventing MALDI-TOF-MS analysis. The remaining 199 were successfully tested by MALDI-TOF-MS and produced spectra. These data were divided into Datasets A and B, with Dataset A serving as the training/initial validation dataset. Optimized models produced from Dataset A were then secondarily tested with Dataset B for generalization to assess their true performance.

**Figure 4:**
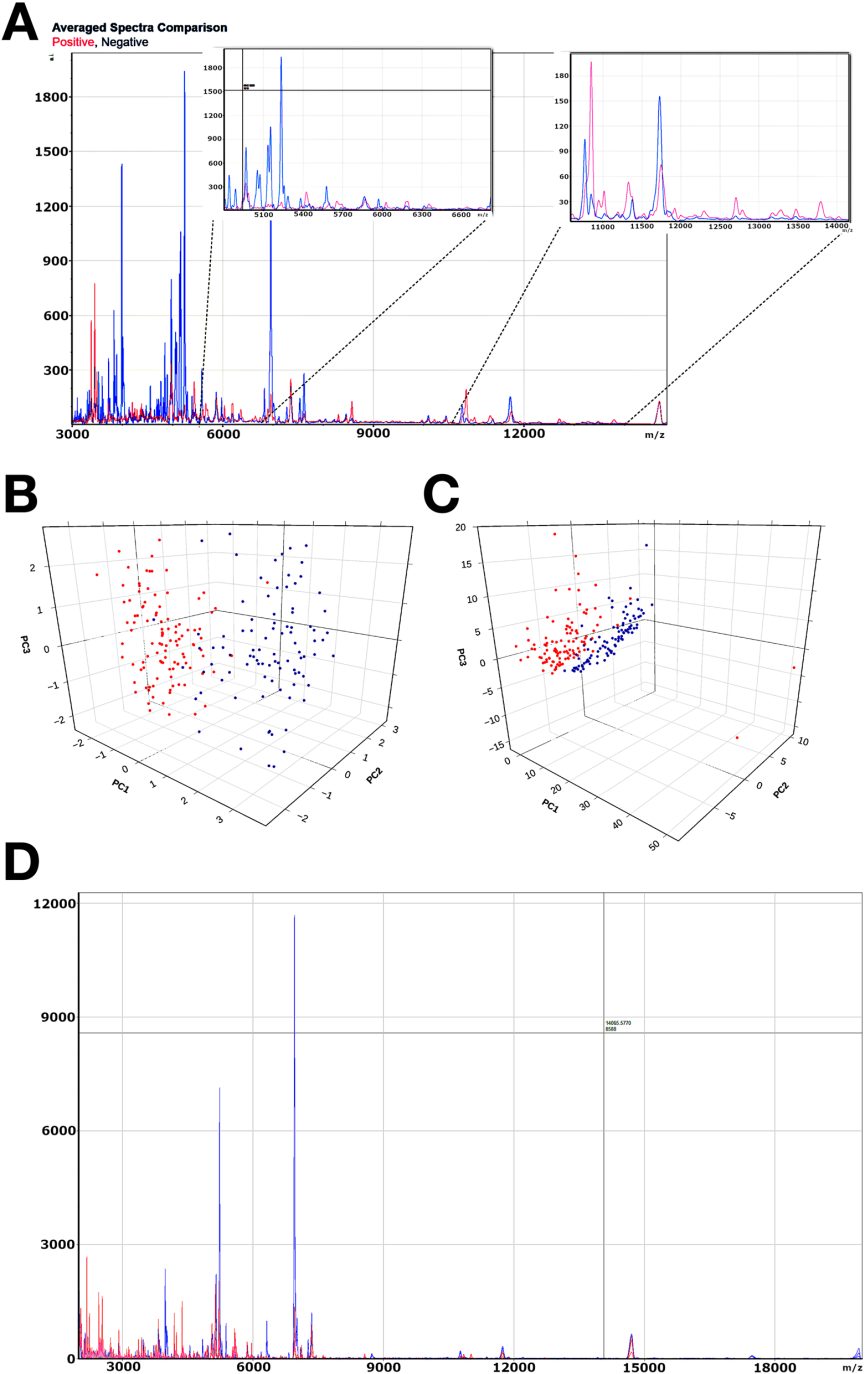
Example MALDI-TOF-MS spectra and PCA analysis of COVID-19 positive vs. negative samples. (**A**) Illustrates the average MALDI-TOF-MS spectra for patients that were SARS-CoV-2 RNA PCR positive (pink) versus PCR negative (blue). Zoomed in regions of interested are also shown. X-axis is mass to charge (m/z) ratio and Y-axis is relative abundance. (**B**, **C**) Show unscaled and scaled PCA, respectively, for the 199 samples (red = positive, blue = negative) tested by the MALDI-TOF-MS method. (**D**) A pair of example (COVID-19 positive vs. negative) patients.

A total of 226 samples were collected with 199 tested by both MALDI-TOF-MS and RT-PCR. Twenty-seven were invalid due to polymer contamination of the sample. For the remaining 199 samples tested, 107 samples were COVID-19 positive (28 asymptomatic and 79 symptomatic) with 92 determined to be negative (Fig. 3). Mean (SD) viral load as measured by RT-PCR cycle threshold (Ct) values was 25.7 (10.9) cycles with a range of 14.5–36.8 cycles. Cycle threshold values were similar between Datasets A and B. Examples of MALDI spectra for COVID-19 PCR positive versus PCR negative patient samples area shown in Fig. 4A,B.

### Data analysis

**Figure 5:**
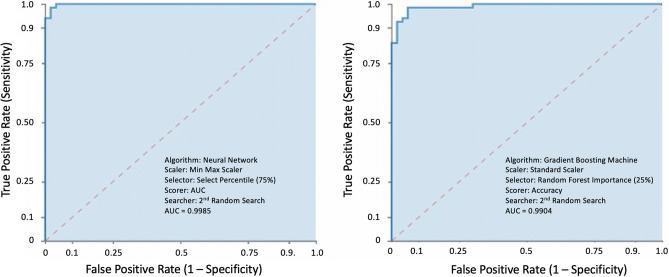
Receiver operator characteristic curves of the top performing ML models. The figure illustrates optimized deep neural network (**A**) and gradient boosting machine (**B**) ML models secondarily tested by Dataset B. For the deep neural network, the ML model used 75% of MS peaks (features) to yield a positive percent agreement of 100% (95% CI 95–100%), and negative percent agreement of 96% (95% CI 86–99%), with an area under the receiver operator curve of 0.9985. In contrast, the Gradient Boosting Machine ML model used only 25% of the MS peaks (features) to yield a positive percent agreement of 99% (95% CI 92–100%) and negative percent agreement of 94% (95% CI 84–99%) with an area under the receiver operator curve of 0.9904.

Prior to ML analysis, an independent principal component analysis was performed (Fig. 4B,C). Thereafter, ML analysis was employed to build and identify the best performing model for the task of distinguishing the COVID positive cases from the negative cases. Figure 4D is an example of a COVID positive patient spectra overlaid over a negative patient spectra. MILO’s automated ML engine was initially trained on a selected subset of the aforementioned data with 82 cases (42 COVID-19 negative and 40 COVID-19 positive) known as Dataset A used for generating the large number of the ML models with initial validation followed by testing each of these models on a secondary generalization Dataset B comprising the remaining 117 cases (50 negative cases and 67 positive cases). MILO produced a total of 379,269 models and identified two models with high performance characteristics within 11 h (Fig. 5). The first is a DNN model with 75% of the total features/MS peaks (487 peaks [range 1993.91–19,590.89 m/z]) that exhibited an accuracy of 98.3% (95% CI 94.0–99.8%), PPA of 100% (95% CI 94.6–100.0%), NPA of 96% (95% CI 86.3–99.5%), with an area under the ROC of 99.9 (95% CI 65.6–100.0). The second model is a GBM model with 25% of the total features/MS peaks (166 peaks [range 2002.72–19,590.89 m/z]) that exhibited an accuracy of 96.6% (95% CI 91.5–99.1%), PPA of 98.5% (95% CI 92.0–100.0%), NPA of 94% (95% CI 83.5–98.8%), with an area under the ROC of 99.0 (95% CI 86.7–100).

## Discussion

The COVID-19 pandemic has created a critical shortage of high performance, high throughput, and rapid solutions for detecting SARS-CoV-2 infection^1,2^. Often, many of these attributes are mutually exclusive, with platforms producing results in < 1 h having lower throughput and/or exhibiting lower clinical sensitivity and specificity compared to their laboratory counterparts^3,4^. Although, as of this paper, supply chains have improved to support testing of symptomatic individuals in a hospital setting, resources remain limited to facilitate and enable widespread rapid screening of asymptomatic individuals necessary for reopening businesses, schools, and other non-hospital settings. Novel molecular solutions have been devised using automated RT-PCR and sequencing platforms. Unfortunately, these mass testing platforms trade high throughput with speed—exhibiting real-world TATs of 24–48 h or even longer.

Our MALDI-TOF-MS approach attempts to address both throughput and speed limitations exhibited by molecular platforms, while maintaining high positive and negative percent agreement. Uniquely, MALDI-TOF-MS does not detect viral RNA and the mass spectra peaks visualized in this technique represents ionizable contents collected from a nasal swab. As illustrated by our study, MALDI-TOF-MS spectra is highly complex, but patterns exist that is differentiable by PCA, but more importantly, by ML techniques that can then generate models that appear to differentiate COVID from non-COVID cases. The use of ML for COVID-19 MALDI-TOF-MS testing of nasal swabs collected in microbiological transport media (i.e., Cary Blair media) has been studied by another group and recently published^10^ with a reported accuracy of 93.9%, and PPA of 95% and NPA of 93%.

Our study differs from Nachigall et al.^10^ in that we directly tested swabs rather than the transport media itself. Additionally, we used readily available saline as a preservative rather than Cary Blair media. Testing personnel consisted of a range of operators from pre-doctoral researchers (e.g., bachelor’s degree), non-laboratory physicians, and clinical laboratory professionals (e.g., licensed clinical laboratory scientists). Personnel received one day of training to achieve sufficient competency to perform testing from sample plating to exporting results to the ML platform. Providing a diverse user base enables this test to be adapted to multiple settings and address personnel considerations defined under the United States Centers for Medicare and Medicaid Services Clinical Laboratory Improvement Amendment (CLIA).

The dependence of ML also required innovative solutions. We accelerated development of ML models with improved percent agreement and eliminated programmer bias by utilizing a clinically validated auto-ML platform to identify optimized models in 11 h. As discussed in this study, as well as others, the manual programming of ML models is both laborious and prone to user bias. These data scientists would need to assess the performance of every feature combination, scaler, and other parameters across all types of ML techniques. Since it is not possible to accomplish this in a reasonable amount of time—especially during the pandemic, data scientists must base their ML development on their experience. The use of MILO auto-ML enables stakeholders to evaluate the performance of every feature combination, scaler, and other parameters across a very large number of ML techniques in about 24 h. In the case of this study, MILO identified two very promising ML models (DNN and GBM), both offering enhanced performance compared to the SVM based model proposed by Nachigall et al.^10^ Notably, MILO’s best SVM (accuracy of 96.6%, and PPA of 98.5% and NPA of 94%), as a comparison, also outperformed the SVM model proposed by Nachigall et al*.* (accuracy of 93.9%, and PPA of 95% and NPA of 93%) which further supports the need of use of such auto-ML platforms within this arena.

**Figure 6:**
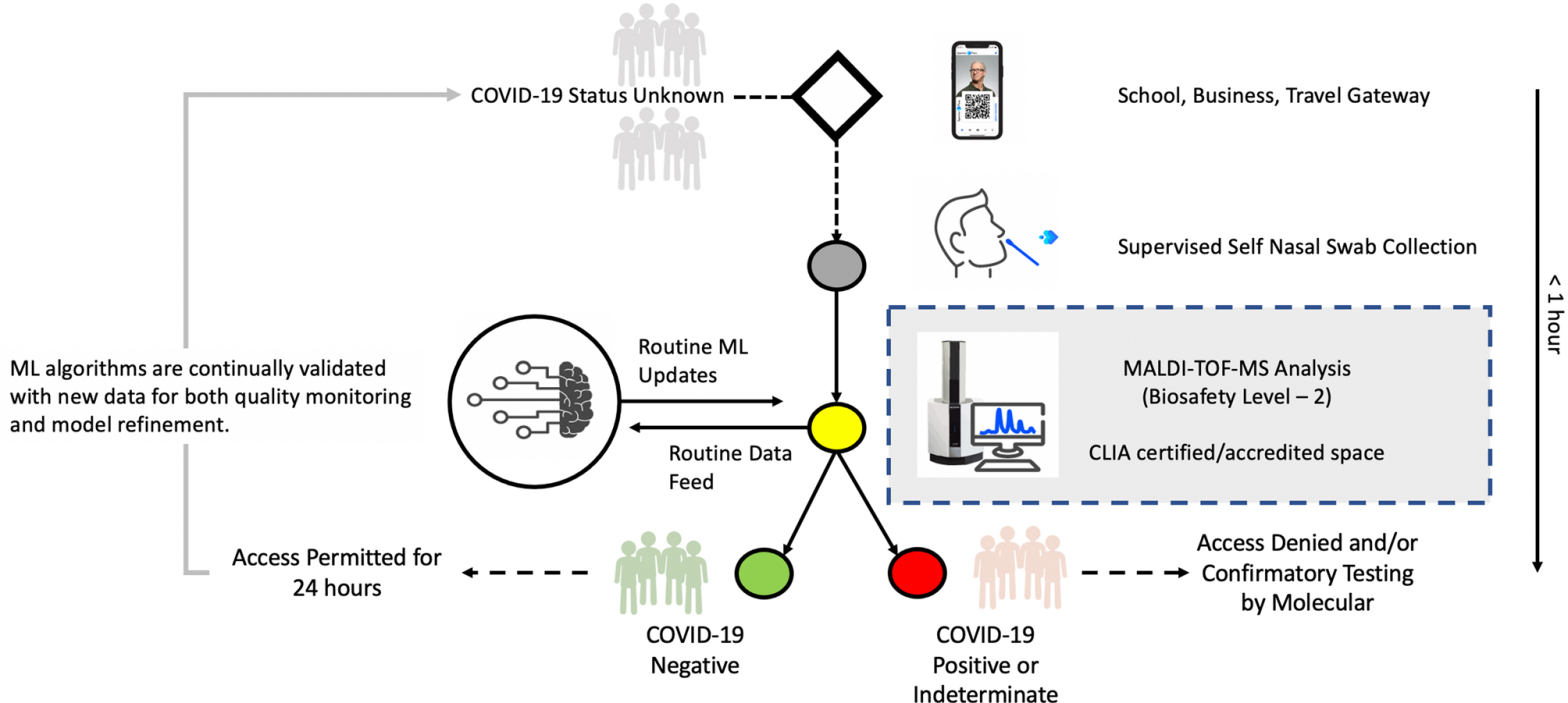
Conceptual model for near patient ML-enhanced MALDI-TOF-MS COVID-19 testing. The Figure outlines the conceptual workflow for our ML-enhanced MALDI-TOF-MS COVID-19 testing method when performed near patient. Individuals with unknown COVID-19 status register via a smart device app which links their identity with a unique quick-response (Q–R) barcode. The Q-R code is paired to the nasal swab specimen which is self-collected under supervision. The sample is tested by MALDI-TOF-MS and mass spectra analyzed by the ML algorithm to report out a COVID-19 result. COVID-19 individuals are allowed entry for 24 h until. COVID-19 positive/indeterminant individuals will be denied entry and/or require follow-up testing by molecular methods. Data from MALDI-TOF-MS is fed routinely to the automated ML platform for both quality assurance and continual refinement of models. Total time from sample collection to result is < 1 h.

Frequent COVID-19 testing is key to reopening schools and businesses until herd immunity is achieved^20^. However, this is not presently sustainable with molecular techniques and rapid antigen tests are still limited by reagent availability, and more importantly, false negative and false positive rates^8,9^. MALDI-TOF-MS combined with ML offers several unique advantages over SARS-CoV-2 molecular and antigen testing. Firstly, the MALDI-TOF-MS technique is rapid, with an analysis time of 20 min. Total turnaround time would be < 1 h and could be accelerated if multiple instruments are available to support random-access testing. Secondly, MALDI-TOF-MS can provide high throughput, with up to 46 samples per run with plus two levels of controls, it has the potential to perform up to 1104 analyses per day per instrument—limited by instrument down time (e.g., preventative maintenance, repairs, etc.) and incoming test volume. In contrast, high throughput commercial RT-PCR platforms described in Table 1 require batch testing for optimal reagent use. As such, these instruments are suitable for reference laboratories that can provide results in 24–72 h. Lastly, the proposed MALDI technique is less dependent on complex supply chains, using readily available bulk chemicals such as acetonitrile, CHCA, ethanol, water, and TFA, whereas molecular assays require a long list of reagents including RNA extraction kits, master mixes, and molecular grade processing plates and pipettes which remain in short supply^6^. Therefore, our approach provides an opportunity to be exploited for re-opening schools and businesses, where testing can be performed with both speed and scale. In order to operationalize this approach, patient registration, specimen collection, testing by MALDI-TOF-MS, and analysis by ML must work as a system rather than be deployed piecemeal. Figure 6 provides a conceptual model for ML-enhanced MALDI-TOF-MS integrated with secure mobile software.

Limitations of this study include the use of frozen clinical specimens as a proof of concept. Proteomic profiling of identified peaks would also be needed to characterize the presence of viral proteins and host response factors. This study was intended to determine if ML-enhanced MALDI-TOF-MS could differentiate between PCR positive COVID-19 patients versus those who tested negative. The study did not evaluate the detection of other coronavirus or influenza like illnesses in the community. Notably, local “shelter in place” have greatly reduced influenza prevalence in the community^21^. Polymer contamination of samples prevented ionization of 27 samples—resulting in an invalid result. Sources of polymer include, but are not limited to the saline itself, specimen collection tube, and the swab. Polymer contamination is unfortunately a common challenge in mass spectrometry^22^.

## Conclusions

Machine learning—enhanced screening of COVID-19 in symptomatic and asymptomatic patients by MALDI-TOF-MS exhibits acceptable positive and negative percent agreement for screening applications. This approach may have great value for testing at satellite laboratories to rapidly screen large numbers of individuals requiring access to businesses, schools and other large facilities. Larger multicenter studies are needed to determine the feasibility of large-scale MALDI-TOF-MS-based COVID-19 detection for workplace screening and further refine ML models that encompass a range of negative COVID-19 vaccinated individuals, vaccinated individuals who still acquire COVID-19, and non-COVID-19 respiratory infectious diseases.

## Data Availability

The datasets generated during and/or analyzed during the current study are available from the corresponding author on reasonable request.
